# The significance of postbypass blood flow model in side to side bypass for moyamoya disease in predicting postoperative cerebral hyperperfusion syndrome

**DOI:** 10.3389/fneur.2024.1484224

**Published:** 2024-11-20

**Authors:** Guiping Wan, Miao Hu, Jin Yu, Can Xin, Tianshu Tao, Wei Quan, Jincao Chen, Jianjian Zhang

**Affiliations:** Department of Neurosurgery, Zhongnan Hospital of Wuhan University, Wuhan, China

**Keywords:** moyamoya disease, surgical procedures, blood flow model, BFM, side to side bypass, cerebral hyperperfusion syndrome (CHS)

## Abstract

**Objective:**

We previously developed the use of side to side (s-s) bypass for the treatment of adult moyamoya disease (MMD) and discovered several kinds of distinct blood flow models intraoperatively, which we observed through indocyanine green-video angiography (ICG-VA). The purpose of this paper was to investigate the correlation between blood flow model (BFM) identified in s-s bypass and the incidence of postoperative cerebral hyperperfusion syndrome (CHS) among patients with MMD.

**Methods:**

We analyzed 166 hemispheres from 153 patients diagnosed with MMD, including 118 hemispheres with s-s bypass and 48 with end to side (e-s) bypass. We categorized the enrolled patients into three pairs of comparison groups based on postoperative CHS (CHS or non-CHS) in s-s bypass, blood flow models (BFM I or BFM II) and surgical approach (s-s bypass or e-s bypass). Patients’ demographics and characteristics were compared between groups.

**Results:**

Among patients who developed CHS, the occurrence of BFM I was more frequent than that of BFM II (0.154 vs. 0.019, *p* = 0.029 < 0.05) and no significant differences were noted in the remaining data. In the group of blood flow models, the proportion of patients with a history of cerebral hemorrhage was higher in BFM II compared to BFM I (0.062 vs. 0.226, *p* = 0.009 < 0.05), and the incidence of severity of ischemia was found to be higher in BFM I than in BFM II (0.774 vs. 0.429, *p* = 0.011 < 0.05), while the postoperative modified Rankin Scale (mRS) score and the Matsushima grade displayed no obvious difference. In comparison with the occurrence of CHS in e-s bypass group (7/48, 0.146), s-s bypass group had no difference (11/118, 0.093; *p* = 0.323 > 0.05), BFM I group showed no discrepancy (10/65, 0.154, *p* = 0.906 > 0.05) while BFM II group was different (1/53, 0.019, *p* = 0.047 < 0.05).

**Conclusion:**

The proportion of postoperative CHS occurring in BFM II during s-s bypass was lower than that in e-s bypass and BFM I. The postbypass blood flow model in s-s bypass may serve as a novel predictor for postoperative CHS.

## Introduction

Moyamoya disease (MMD) is a chronic ischemic cerebrovascular disease characterized by progressive stenosis and/or occlusion of the end of unilateral or bilateral internal carotid artery and the initial segment of its branches, accompanied by abnormal smoke-like vascular network in the skull base. Therefore, MMD, also known as spontaneous occlusion of circle of Willis, is the main cause of ischemic and hemorrhagic stroke (1, 2). Since Donaghy and Yasargil (3) first proposed microvascular anastomosis as a treatment for middle cerebral artery occlusion, the superficial temporal artery (STA) to middle cerebral artery (MCA) anastomosis has become a progressively utilized method for treating MMD. However, postoperative complications, particularly cerebral hyperperfusion syndrome (CHS), continue to pose significant challenges following direct revascularization procedures. Postoperative CHS can cause neurological symptoms, including transient and permanent neurological defects, and with the increase of intracranial vascular pressure, it can lead to cerebral vascular rupture resulting in cerebral hemorrhage (4, 5).

To date, numerous articles have reported on various devices and theories aimed at predicting and preventing the onset of CHS associated with direct revascularization. These include tools such as transcranial Doppler (TCD), single-photon emission computed tomography (SPECT), ICG-FLOW 800, and micro-Doppler ultrasonography (MDU). Additionally, improvements in surgical techniques have been explored, such as reducing the lumen of the donor blood vessel through methods like electrocoagulation, burning, or suturing, increasing the degree of vascular turning, or simultaneously separating the parietal and frontal branches of the STA (6–12). Zhang et al. (13), furthering the work of Lang et al. (14) reintroduced the s-s bypass for MMD and advocated for its adoption as a standard surgical approach. They posited that the STA could autonomously adjust the blood flow into the brain in response to intracranial blood flow demands, potentially reducing the incidence of CHS while preserving the distal branch of the STA. However, to date, no clinical trials have been able to confirm this purported self-regulation mechanism.

During our s-s bypass surgeries, we observed two distinct blood flow models. This paper aims to investigate whether the s-s bypass can reduce the incidence of the occurrence of CHS in comparison to the e-s bypass. Furthermore, we explore the correlation between the blood flow model (BFM) and the risk of CHS. Additionally, we endeavor to delve into the formation and prognosis of BFM, offering insights into the underlying mechanisms and potential therapeutic implications.

## Methods

### Patient and inclusion criteria

This study encompassed 166 hemispheres across 153 patients, with all participants following the guidelines established by the Research Committee on Moyamoya Disease of the Ministry of Health, Labor, and Welfare of Japan (15). The observation period spanned from September 2022 to May 2024. In addition to the basic inclusion criteria, the patient cohort for our study needed to meet the following specific requirements: (1) postoperative magnetic resonance angiography (MRA) or computed tomography angiography (CTA) should have been conducted to confirm the patency of the anastomosis; patients with occluded bypass vessels were excluded from the study; (2) included patients must have undergone computed tomography (CT) brain scans and magnetic resonance imaging-diffusion weighted imaging (MRI-DWI) to assess cerebral hemorrhage and acute infarction; (3) for patients with no abnormalities detected in postoperative CT and MRI-DWI, hemodynamic changes were evaluated using SPECT or computed tomography perfusion (CTP). Any hemispheres not fulfilling these criteria were excluded from our research.

The study protocol was approved by the Ethics Committee of the Zhongnan Hospital of Wuhan University and was conducted in compliance with the Declaration of Helsinki, as revised in 1983. Written informed consent was obtained from all participants involved in the study.

### Patients’ management and surgical procedures

Prior to both the s-s and e-s bypass procedures, patients typically received an intravenous infusion of 1,000 mL of compound dextran the night before surgery. This was done to ensure vessel filling, reduce the risk of cerebral infarction, and facilitate intraoperative manipulation. During the anesthesia process, intravenous anesthesia was employed, with strict control of systolic blood pressure to approximately 120 mmHg to prevent undue fluctuations. Additionally, partial pressure of carbon dioxide (PCO_2_) levels were managed within the normal range to avoid the risk of cerebral infarction that could result from aggressive ventilation. Postoperatively, maintaining fluid balance was a priority, with the target systolic blood pressure being maintained between 110 and 130 mmHg. All bypass operations were performed by a neurosurgeon. The specific surgical procedures before and after s-s and e-s anastomosis were completely referred to our previous literature and we tried to choose recipient vessels with a diameter similar to that of donor vessels, controlling the size of the anastomotic stoma to be about 1.2 times the diameter of the recipient blood vessel (13).

The patient was positioned supine with the head turned to the contralateral side to facilitate access to the surgical field. Doppler ultrasonography was employed to meticulously map the trajectories of the frontal and parietal branches of STA. After identifying the appropriate STA branch, the temporalis muscle was incised down to the bone and reflected anteriorly, creating a 7 by 8 centimeter exposure at the junction of the temporal and frontal bones. This dissection provided adequate access to the MCA cortical branches in the region surrounding the sylvian fissure. Once the cortical surface was adequately exposed, ICG-VA was utilized to carefully select a recipient artery suitable for anastomosis based on the diameter and the hemodynamic characteristics. MDU was then applied to assess the velocity and direction of blood flow of vessels. In the majority of cases, a typical arteriotomy required only 3 to 4 sutures per arterial wall. Following the completion of the direct anastomosis, ICG-VA was promptly repeated to verify the patency of the bypass and MDU was utilized once more to probe the velocity and direction.

**Figure 1:**
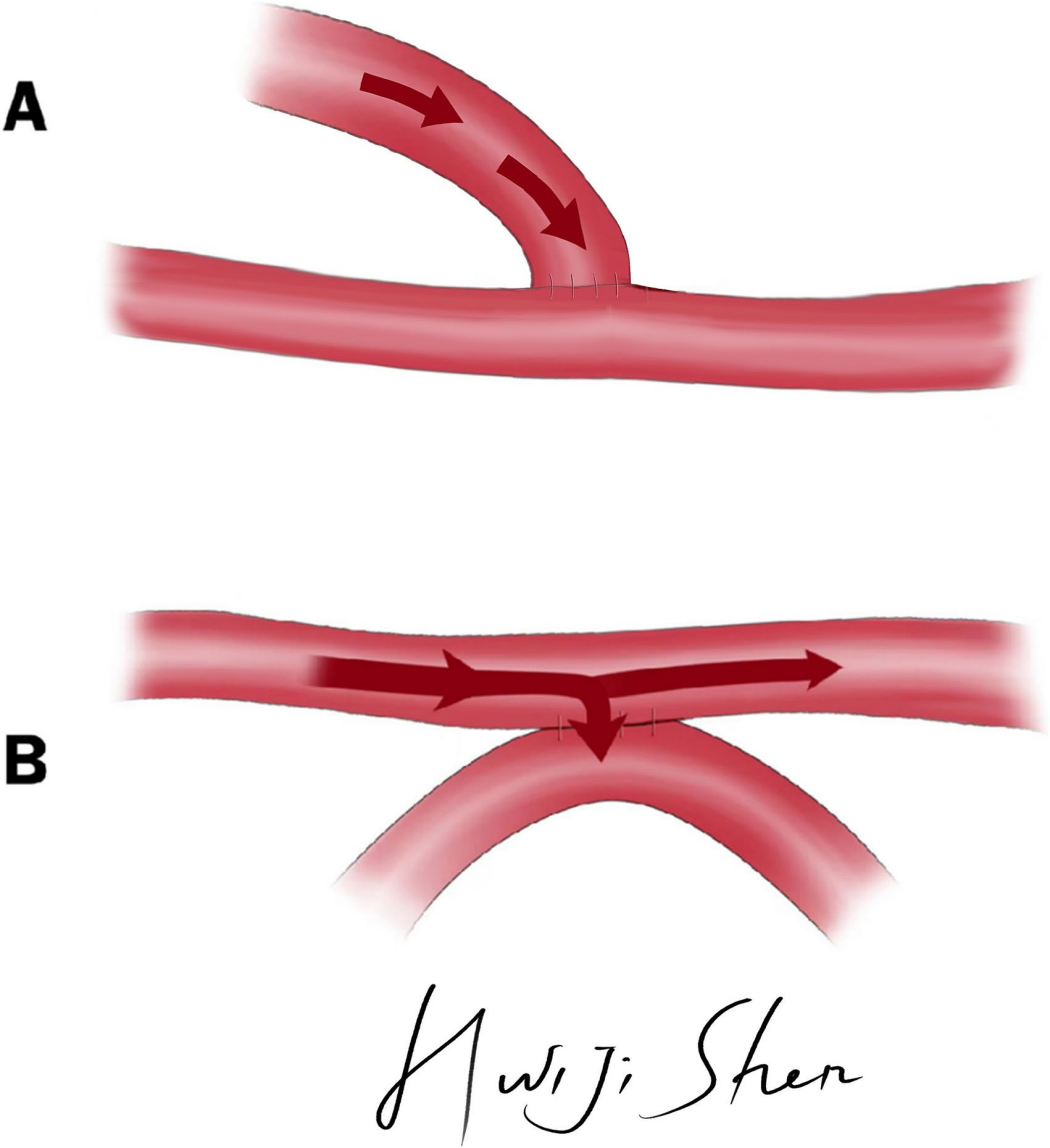
The illustration of e-s bypass and s-s bypass. **(A)** Illustration of e-s bypass. **(B)** Illustration of s-s bypass.

The e-s bypass and s-s bypass was illustrated in Figure 1.

### Device setup

During our trial, we had used three kinds of devices: micro-Doppler ultrasonography (MDU), indocyanine green-video angiography (ICG-VA) and Flow800, which have been demonstrated the feasibility, safety, and effectiveness in the operation of MMD (6, 8, 16). The details are as follows:

MDU: Prior to anastomosis, the MDU probe was utilized to meticulously measure the velocity direction and magnitude of the STA on three separate occasions to minimize potential inaccuracies. Postoperatively, the distal STA, within a proximity of 1 centimeter to the anastomotic site, was re-examined using an identical procedure. The probe was consistently positioned at a 45-degree angle relative to the blood vessel and the MDU device we utilized was the DWL Doppler BOX, a product registered by German Compumedics.
ICG-VA: ICG-VA was performed by using a surgical microscope (ZEISS KINEVO 900). Patients were administered 15 mL of ICG solution (containing 5 mg of ICG) intravenously and the ICG-VA image would appear within several seconds after the injection.
Flow800: The Flow800 software analysis was based on the aforementioned ICG-VA images and was also facilitated using the same surgical microscope (ZEISS KINEVO 900). This software provided further detailed hemodynamic assessment, augmenting the precision of our surgical interventions.

### The definition of blood flow model

**Figure 2:**
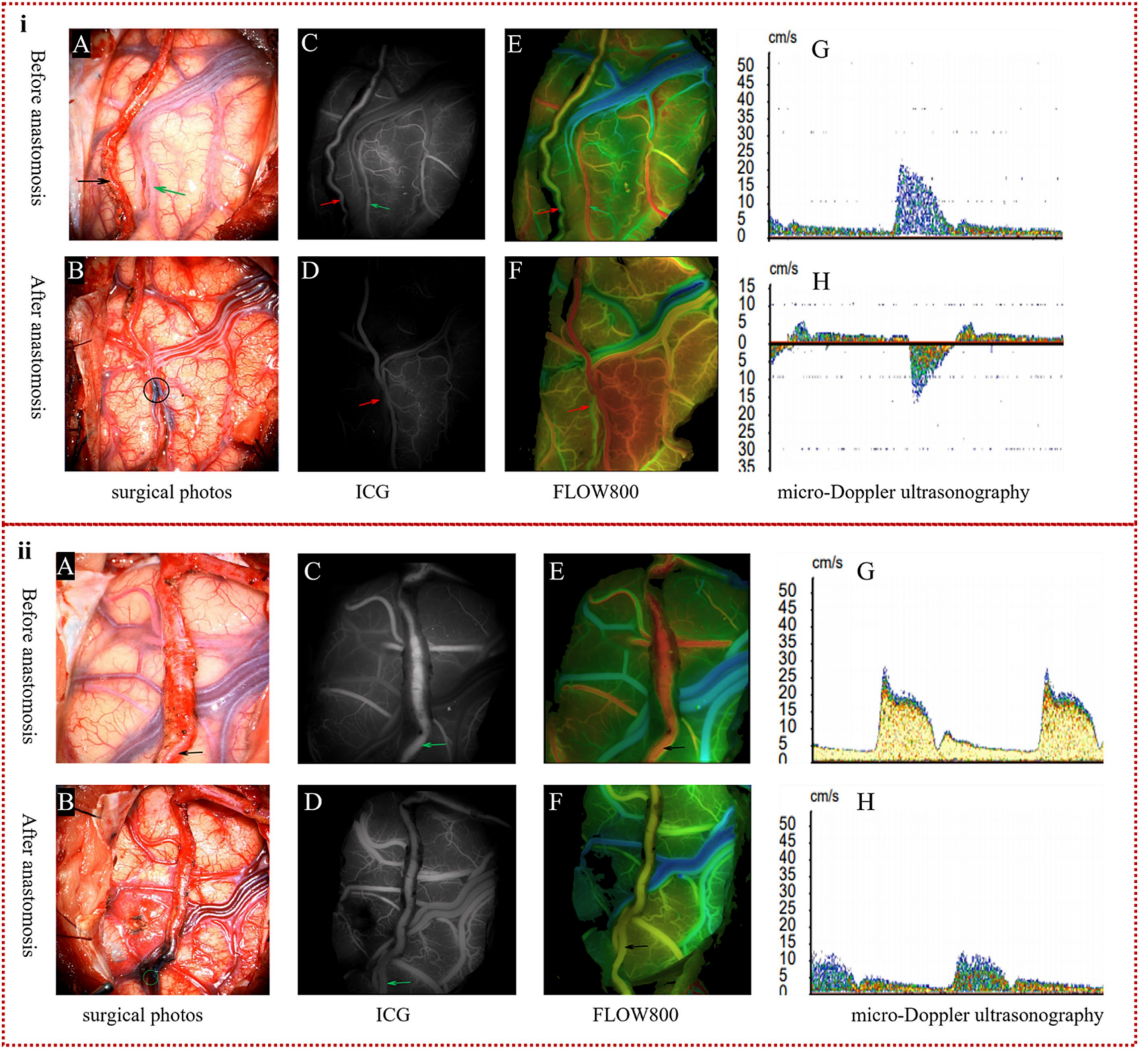
The definition of two kinds of blood flow models. **(i)** Specification of BFM I: in the surgical images, the donor artery (indicated by the black arrow in **A**) and the recipient artery (marked by the green arrow in **A**) were successfully anastomosed by s-s bypass, as shown by the black circle **(B)**. ICG-VA clearly displayed the donor artery (red arrow in **C**) and the recipient artery (green arrow in **C**) prior to anastomosis; however, the distal STA (red arrow in **D**) was not visible post-s-s anastomosis during the arterial phase. Flow800 imaging confirmed continuous color representation of the STA (red arrow in **E**) before surgery, but a distinct color discontinuity was observed around the anastomosis site (red arrow in **F**) after the procedure. MDU detected a unidirectional flow in the STA **(G)** before anastomosis, which changed to bidirectional flow **(H)** after the anastomosis. **(ii)** Definition of BFM II: in the surgical imagery, the intact parietal branch of the STA served as the donor vessel, indicated by the black arrow **(A)**, and an excellent s-s anastomosis is depicted within the green circle **(B)**. ICG-VA and Flow800 assessments revealed a clear depiction of the STA (green arrows in **C,D**), with a continuous color representation (black arrows in **E,F**) observed both pre- and postoperatively. MDU confirmed that although the velocity of the STA was lower than preoperative values, the direction of blood flow remained unchanged, as shown in **(G,H)**.

We categorized the blood flow models (BFMs) observed during our surgeries into two distinct types, BFM I and BFM II, with the following defining characteristics:

BFM I: Following the s-s anastomosis, ICG-VA confirmed that blood flow was unobstructed, but there was a significant delay observed in the distal STA, marked by oscillating blood flow patterns. Simultaneously, Flow800 qualitatively demonstrated a discontinuity in the color stage between the proximal and distal STA, in contrast to the continuity present before the anastomosis. Using MDU, we detected a transition from preoperative unidirectional to bidirectional velocity in the distal STA, as illustrated in Figure 2i. Insights from ICG-VA are provided in Supplementary Video S1.
BFM II: After the anastomosis, ICG-VA confirmed uninterrupted blood flow, with no delays detected in the distal STA. Flow800 showed continuous color stages between the proximal and distal STA, indicating a consistent blood flow pattern. MDU analysis revealed that the distal STA maintained its unidirectional flow, aligning with the preoperative direction, albeit at a reduced velocity as compared to preoperative assessments, as depicted in Figure 2ii. Comprehensive visualization of the ICG-VA for this scenario is presented in Supplementary Video S2.

### The classification of ischemic degree

**Figure 3:**
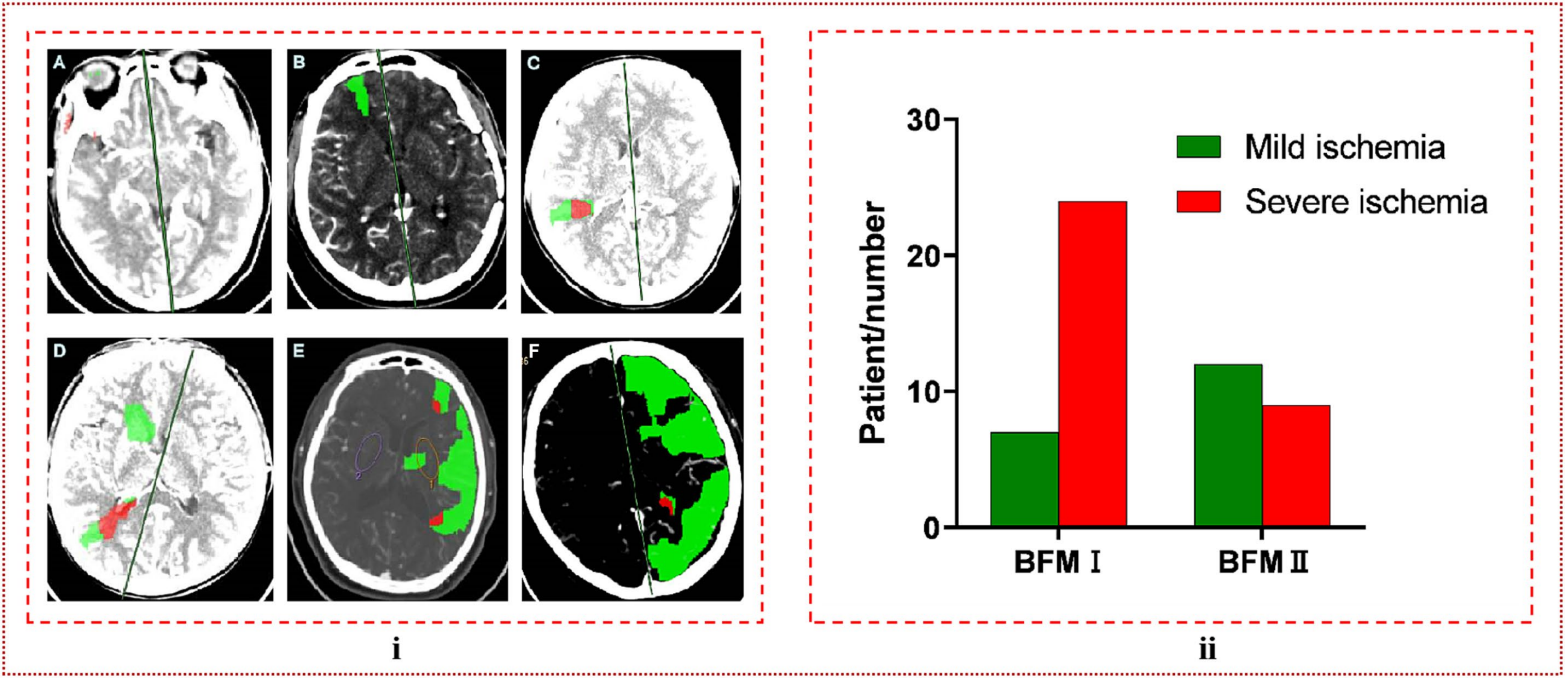
**(i)** The classification of ischemic degree. **(A)** No abnormalities were detected, which is referred to as mild ischemia. **(B)** The green coloration was limited to a single brain lobe, a condition we designate as moderate ischemia. **(C)** The presence of a red region signified severe ischemia. **(D–F)** When red or green regions extended beyond the boundaries of a single brain lobe, both scenarios were categorized as severe ischemia. **(ii)** In comparing the ischemic severity between BFM I and BFM II, it was observed that the rate of severe ischemia in BFM I was significantly higher than in BFM II, with a clear and statistically significant difference observed (24/31, 0.774 vs. 9/21, 0.429, *p* = 0.011 < 0.05).

We simply divided the ischemic degree into two kinds of forms: severe ischemia and mild ischemia, based on the surgical side of the cerebral hemisphere, as specified below:

We selected 52 patients who had previously undergone bypass surgery on the contralateral side or were diagnosed with unilateral MMD. The patients’ perfusion was assessed by CTP before s-s bypass, and the post-processing of CTP data allowed us to visualize the scope and extent of cerebral ischemia in comparison to the contralateral brain. In this comparison, green was indicative of areas where the mean transit time (MTT) was more than 50% greater than the contralateral side and the cerebral blood volume (CBV) exceeded 2 mL/100 g. Conversely, red represented regions where the MTT was over 50% higher than the opposite side and the CBV was less than 2 mL/100 g. Mild ischemia was defined as instances where the CTP scan revealed no significant abnormalities, or when the green area was limited to a single brain lobe without being significantly extensive. Conversely, severe ischemia was identified by the presence of red areas, or when the green area expanded beyond the boundaries of a single brain lobe. Additional details and visual representations of these classifications are presented in Figure 3i.

### The specification of CHS

In our study, the diagnosis of CHS was contingent upon meeting two specific criteria:

- Within the two-week period following bypass surgery, patients presented with clinical symptoms suggestive of CHS, such as contralateral limb weakness, seizures, speech disorders, headaches, and dizziness.
- Patients experienced immediate cerebral hemorrhage during the anastomosis process, with the exclusion of any surgical errors as the cause. Moreover, CT scans confirmed the presence of cerebral hemorrhage, or SPECT/CTP showed a significant increase in regional cerebral blood flow (rCBF) around the anastomotic site, qualitatively marked by a pronounced focal enhancement in the perfusion stage.

The development of CHS post-bypass surgery was concluded only when a patient met both of these criteria. All imaging assessments were performed by two experienced neurosurgeons who were unaware of the experimental conditions, ensuring the objectivity and reliability of the outcomes.

### Statistical analysis

SPSS Statistics Desktop version 25.0 (IBM Corp.) was used for all the statistical calculations. Differences in the distribution of continuous variables were analyzed by using the Shapiro–Wilk (SW) tests and the final results were presented by medians and interquartile ranges (IQRs), as well as the categorical variables were analyzed in contingency tables with the chi-square test. Statistical significance was set at *p* < 0.05.

## Results

### Comparison between CHS group and non-CHS group in s-s bypass

**Figure 4:**
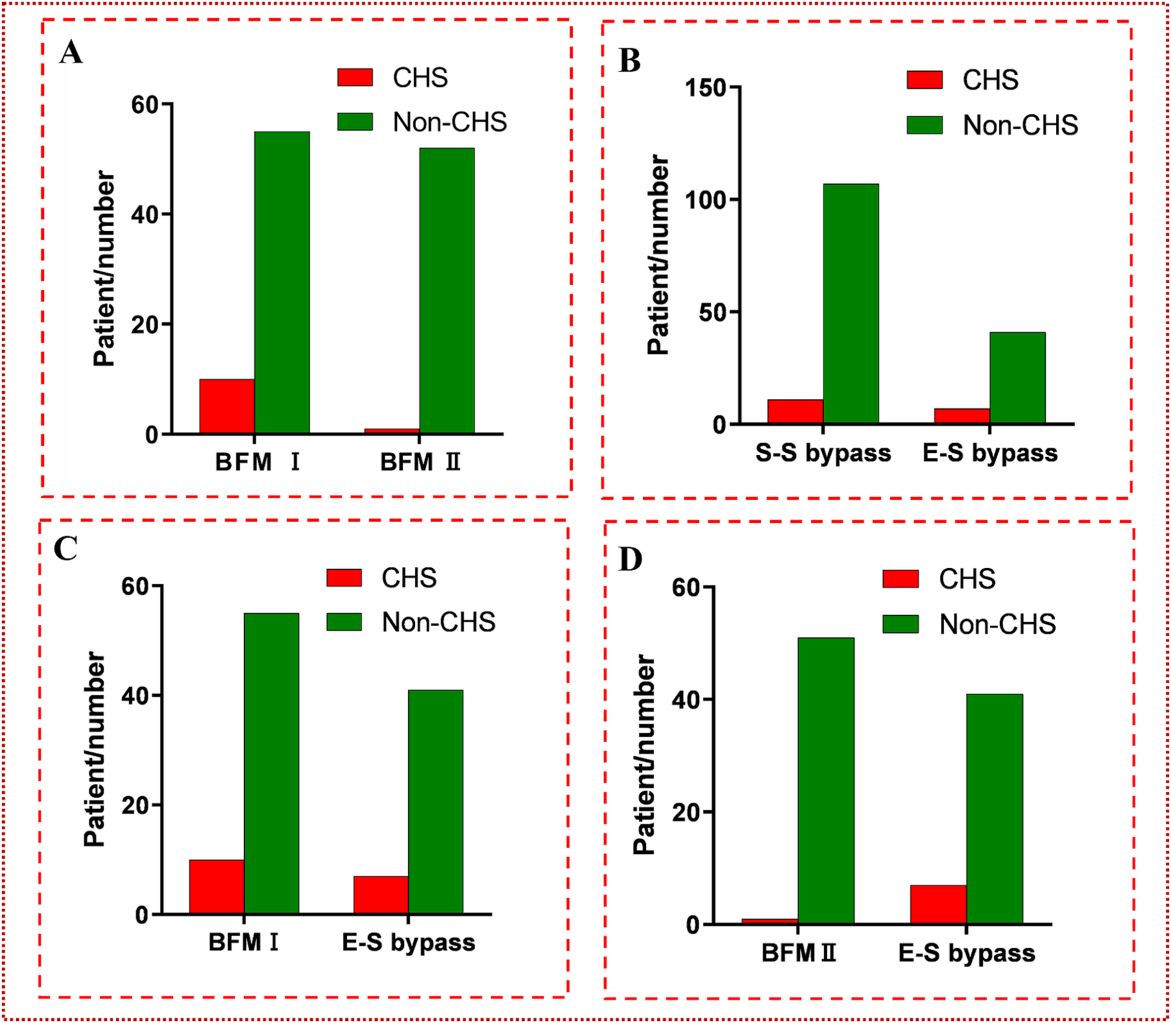
**(A)** The comparison between BFM I and BFM II. The incidence of CHS in BFM I (10/65, 15.4%) was much higher than the incidence of CHS in BFM II (1/53, 1.9%) (*p* = 0.029 < 0.05). **(B)** The comparison between s-s bypass and e-s bypass. No difference was found between them (7/48, 14.6% vs. 11/118, 9.3%; *p* = 0.323 > 0.05). **(C)** The comparison between BFM I and e-s bypass. No difference was found at them (7/48, 14.6% vs. 10/65, 15.4%; *p* = 0.906 > 0.05). **(D)** The comparison between BFM II and e-s bypass. Obviously distinction was shown (1/53, 1.9% vs. 7/48, 14.6%; *p* = 0.047 < 0.05).

In the cohort of patients who underwent s-s bypass surgery, CHS was observed in 11 out of 118 hemispheres, with a patient demographic that included 61 females (51.7%) and 57 males (48.3%). The foundational data of these cases were presented in Table 1. Upon conducting analysis, no significant disparities were detected between patients who developed CHS post-s-s anastomosis and those who did not, across a spectrum of factors including demographic attributes, such as sex and age; comorbidities like hypertension and diabetes, history of cerebral infarction or hemorrhage, the laterality of the surgery, the mRS score on admission, and the Suzuki stage. However, the incidence of CHS was higher in the BFM I group, with 10 out of 65 cases (15.4%) experiencing CHS, compared to only 1 out of 53 cases (1.9%) in the BFM II group (*p* = 0.029), as shown in Figure 4A. An exhaustive profile of the patients was detailed in Table 2.

**Table 1:** Characteristics of the CHS patients in s-s bypass and e-s bypass.

No.	Sex	Age (yeras)	Onset	Symptom	Suzuki stage	Comorbidities	Methods	Surgical side	BFM	Complications	Admission mRS score	Postoperative mRS score
1	M	49	Infarction	R-limbs weakness	III	Hypertension	s-s	R	I	Motor aphasia	3	3
2	M	64	Ischemic	L-limbs numbness	V	Hypertension, diabetes	s-s	L	I	Seizure attack	1	1
3	F	59	Ischemic	—	V	Hypertension	s-s	R	I	Cerebral hemorrhage with contralateral limbs weakness	0	2
4	F	41	Infarction	Memory decline	IV	Hypertension	s-s	R	I	Seizure attack	1	2
5	F	52	Infarction	—	IV		s-s	R	I	Motor aphasia	0	1
6	F	54	Ischemic	Dizziness and headache	V	Hypertension	s-s	L	I	Cerebral hemorrhage with contralateral limbs weakness and speech disorder	1	3
7	M	36	Ischemic	—	IV	Hypertension	s-s	L	II	Receptive aphasia	0	1
8	F	59	Ischemic	Dizziness	IV	—	s-s	R	I	Contralateral limbs weakness	1	2
9	F	54	Ischemic	Both limbs weakness	III	—	s-s	R	I	Cerebral hemorrhage during operation	0	1
10	F	53	Ischemic	Transient loss of consciousness	III	—	s-s	L	I	Motor aphasia	1	1
11	M	36	Ischemic	Headache	III	—	s-s	R	I	Cerebral hemorrhage during operation	1	1
12	F	47	Ischemic	Dizziness	IV	Diabetes	e-s	L	—	Motor aphasia	1	2
13	F	53	Cerebral hemorrhage	L-limbs weakness	V	—	e-s	R	—	Receptive aphasia	1	2
14	M	47	Ischemic	Headache	V	Hypertension, Diabetes	e-s	L	—	Seizure attack	1	2
15	M	45	Ischemic	Transient aphasia	IV	Hypertension	e-s	R	—	Headache	1	2
16	M	56	Ischemic	—	III	Hypertension	e-s	R	—	Contralateral limbs weakness	0	2
17	F	52	Ischemic	Recurrent TIA	III	—	e-s	L	—	Cerebral hemorrhage with contralateral limbs weakness	1	3
18	F	37	Ischemic	—	IV	—	e-s	L	—	Motor aphasia	1	2

**Table 2:** The patients’ basic information between CHS group and non-CHS group in s-s bypass.

	CHS (*n* = 11)	Non-CHS (*n* = 107)	*p*-value
Age, years	50.6 (43.0–57.0)	49.5 (41.0–59.0)	0.747
Sex			0.405
Female	7 (63.6)	54 (50.5)	
Male	4 (36.4)	53 (49.5)	
Surgical side			0.474
Left	4 (36.4)	51 (47.7)	
Right	7 (63.6)	56 (52.3)	
Hypertension	6 (54.5)	49 (45.8)	0.580
Diabetes	1 (9.1)	21 (19.6)	0.654
Preoperative cerebral infarction	3 (27.3)	19 (17.8)	0.715
Preoperative cerebral hemorrhage	0	16 (15.0)	0.359
Admission mRS score	0.91 (1–1)	0.82 (0–1)	0.770
Suzuki stage			0.455
II	0	6 (5.6)	
III	4 (36.4)	32 (29.9)	
IV	4 (36.4)	24 (22.4)	
V	3 (27.3)	45 (42.1)	
BFM			0.029 < 0.05
I	10 (90.9)	55 (64.6)	
II	1 (9.1)	52 (35.4)	

CHS, cerebral hyperperfusion syndrome; mRS, modified Rankin Score; BFM, blood flow model. *p* < 0.050 were considered significant. Values are shown as number (%) or median (interquartile ranges) unless indicated otherwise.

### Comparison between BFM I and BFM II in s-s bypass

**Figure 5:**
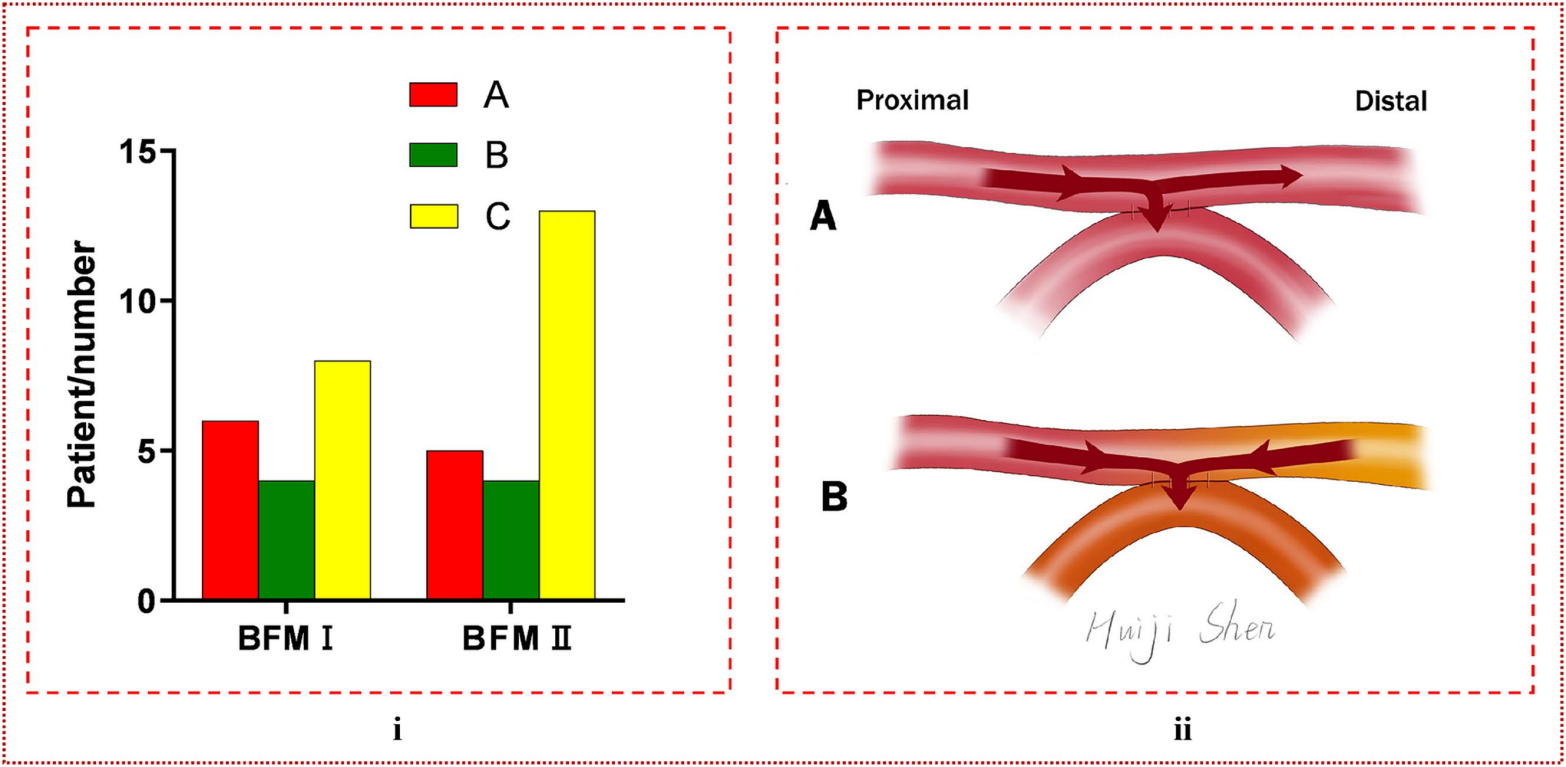
**(i)** The comparison of Matsushima grade between BFM I and BFM II. No difference was found between them (*p* = 0.640 > 0.05). **(ii)** The manifestation of blood flow model. **(A)** Blood can be diverted from the distal STA, with a portion of it flowing into the brain, thereby reducing the incidence of CHS, and is termed BFM II. **(B)** Blood from both the proximal and distal segments of the STA enters the intracranial space, potentially leading to the occurrence of CHS, and is designated as BFM I. In this visualization, red represents blood from the proximal STA, yellow indicates blood from the distal STA, and orange denotes the mixture of these two blood types entering the recipient artery.

The analysis comparing the preoperative fundamental details between BFM I and BFM II disclosed that the proportion of patients with a history of cerebral hemorrhage was higher in BFM II compared to BFM I, while the patients’ other basic information and postoperative mRS score showed no difference, which could be seen in Table 3. In the subset of 40 hemispheres that underwent repeat digital subtraction angiography (DSA) subsequent to the s-s bypass, the BFM I group comprised 18 hemispheres with Matsushima grades distributed as A: 6, B: 4, and C: 8 while the BFM II group included 22 hemispheres with the following distribution: A: 5, B: 4, and C: 13. No significant differences were noted in this comparison (*p* = 0.640 > 0.05), as shown in Figure 5i. In our research, 52 of 118 hemispheres with s-s bypass experiencing CTP examination were classified to mild ischemia and severe ischemia two groups. The BFM I group included 7 hemispheres with mild ischemia and 24 with severe ischemia. Comparatively, the BFM II group had 9 hemispheres with mild ischemia and 12 with severe ischemia. A clear correlation was established between the degree of ischemia and the BFM manifestations (0.774 vs. 0.429, *p* = 0.011 < 0.05), which was displayed in Figure 3ii.

**Table 3:** The patients’ basic information between BFM I group and BFM II group in s-s bypass.

	BFM I (*n* = 65)	BFM II (*n* = 53)	*p*-value
Age, years	48.9 (43.0–57.5)	50.4 (43.0–58.0)	0.463
Sex			0.335
Female	31 (47.7)	30 (56.6)	
Male	34 (52.3)	23 (43.4)	
Surgical side			0.630
Left	29 (44.6)	26 (49.1)	
Right	36 (55.4)	27 (50.1)	
Hypertension	30 (46.2)	25 (47.2)	0.912
Diabetes	13 (20.0)	9 (17.0)	0.675
Preoperative cerebral infarction	15 (23.1)	7 (13.2)	0.171
Preoperative cerebral hemorrhage	4 (6.2)	12 (22.6)	0.009 < 0.05
Suzuki stage			0.117
II	2 (3.1)	4 (7.5)	
III	21 (32.3)	15 (28.3)	
IV	11 (16.9)	17 (32.1)	
V	31 (47.7)	17 (32.1)	
Admission mRS score	0.83	0.83	0.996
Postoperative mRS score	0.82	0.75	0.727

mRS, modified Rankin Score; BFM I, blood flow model I; BFM II, blood flow model II. *p* < 0.050 were considered significant. Values are shown as number (%) or median (interquartile ranges) unless indicated otherwise.

### Comparison between s-s bypass and e-s bypass

In the group of 48 hemispheres that underwent e-s bypass, CHS was observed postoperatively in 7 cases, as detailed in Table 1. When compared to the incidence of CHS in the s-s bypass group, no significant difference was noted (7/48, 14.6% vs. 11/118, 9.3%; *p* = 0.323 > 0.05). A subsequent comparison between the e-s bypass group and BFM I group likewise revealed no significant difference in the occurrence of CHS (7/48, 14.6% vs. 10/65, 15.4%; *p* = 0.906 > 0.05). However, a notable distinction was observed when comparing e-s bypass to BFM II, with a significantly lower incidence of CHS in the latter group (7/48, 14.6% vs. 1/53, 1.9%; *p* = 0.047 < 0.05). More detailed data can be seen in Figure 4.

### Representative case

**Figure 6:**
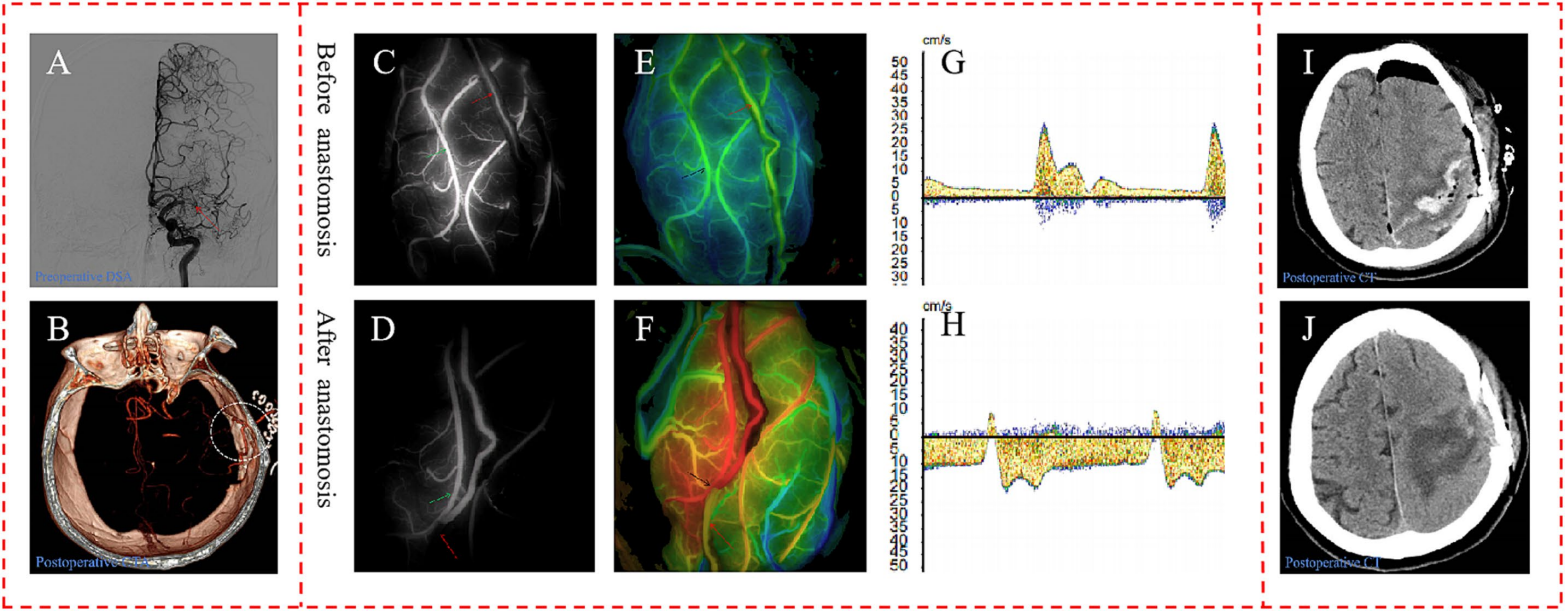
Representative case. **(A)** Preoperative DSA showed initial segment occlusion of the left MCA with abnormal smoke-like vessels (red arrow). **(B)** Postoperative CTA confirmed the patency of the bypass, with the anastomotic stoma highlighted within a white circle. The ICG-VA demonstrated the patency of the bypass and a delay in the distal donor vessel, in which the donor vessel was indicated by the red arrow, and the recipient vessel is marked with a green arrow **(C,D)**. In the Flow800 imaging, there was an abrupt change in the color of the distal STA when compared to the preoperative state, as indicated by the red arrow **(E,F)**. And MDU showed the direction of distal STA was bidirectional, compared to the unidirectional flow observed preoperatively **(G,H)**. The initial postoperative CT scan on the first day after surgery displayed evidence of cerebral hemorrhage **(I)**. By the second week post-operation, the hematoma had been nearly entirely absorbed, indicating a recovery in the patient’s condition **(J)**.

A 54-year-old female patient, presenting with symptoms of dizziness and headache, was admitted to our hospital and DSA confirmed the diagnosis of MMD (Figure 6A). Intraoperatively, indocyanine green-video angiography (ICG-VA) indicated no delay in the donor vessel after it was dissected free from the surrounding tissues and we selected a recipient vessel with a diameter closely matching that of the STA (Figure 6C). However, a delay was observed in the distal STA following the s-s anastomosis (Figure 6D). Utilizing Flow800, we detected a discontinuity in the color of the STA compared to the pre-surgical state, along with a distinct area of blood flow filling around the recipient vessel (Figures 6E,F). Micro-Doppler ultrasonography (MDU) was then reapplied, confirming the bidirectionality of blood flow in the distal STA within 1 cm of the anastomotic stoma, as opposed to the preoperative unidirectional flow (Figures 6G,H). Prior to skull closure, a sudden bleeding area was identified distant from the bypass site. Immediate action was taken to evacuate the hematoma and constrict the proximal donor vessel to decrease the blood flow into the skull. Postoperatively, the patient exhibited contralateral limbs weakness and speech disturbances, with the CT scan revealing cerebral hemorrhage (Figure 6I), while CTA confirmed the patency of the anastomosis (Figure 6B). After a series of conservative treatments, including stringent control of blood pressure, the patient’s speech gradually improved, and the muscle strength in her right limbs eventually reached grade IV. A CT reexamination 2 weeks postoperatively indicated near-complete resorption of the hematoma (Figure 6J), and the patient was subsequently discharged for rehabilitation.

## Discussion

This study demonstrated that there was no significant difference in the proportion of CHS occurring between the e-s and s-s bypass techniques. However, within the s-s bypass method, the probability of developing CHS in BFM II was lower than that in BFM I and the e-s bypass. Additionally, our research suggested a potential correlation between the formation of the two distinct blood flow models and the degree of cerebral ischemia.

As an innovative surgical intervention for the treatment of MMD, the literature on the specific dynamics of s-s anastomosis in comparison to e-s anastomosis, particularly regarding blood flow models, was quite limited. Zhang et al. (13) have proposed that the self-regulation of the s-s bypass presents in three distinct forms:

- The distal STA can divert a small amount of blood, directing the majority towards the intracranial circulation.
- The intracranial bed can draw a significant volume of blood from both the proximal and distal STA.
- If the intracranial ischemia is not severe, the distal STA can shunt the majority of the blood, potentially averting the development of CHS.

This conceptualization of the s-s bypass suggested a level of adaptability in blood flow regulation that could offer clinical advantages over traditional e-s anastomosis techniques. However, further empirical investigation is needed to validate these theoretical benefits and to elucidate the nuances of blood flow dynamics following s-s anastomosis in the context of MMD treatment. In our research, we simply divided it to two different models based on clinical manifestation: one was that the distal STA can shunt blood named BFM II, and the other was that the blood of distal STA can flew into the intracranial through the anastomosis which cannot shunt blood referred to as BFM I. These two models were illustrated in Figure 5ii.

We hypothesized that in cases where the ischemic condition in the hemisphere was particularly severe, the M4/M5 segment of the MCA might also be under extreme ischemic stress due to intracranial blood stealing. Upon the establishment of an s-s anastomosis, the M4/M5 segment could draw a significant amount of blood into the intracranial space, not only from the proximal but also from the distal STA. This phenomenon was unique to the s-s anastomosis because the distal end of the donor blood vessel remained intact. The influx of excessive blood into the intracranial compartment could potentially elevated the risk of CHS incidence. For patients exhibiting BFM II, characterized by milder brain ischemia, it was likely that the majority of blood flow would follow the preserved distal vessels. Consequently, with less blood entering the brain, the likelihood of CHS occurrence would be reduced. This hypothesis was corroborated by our research, which demonstrated a higher rate of severe ischemia in BFM I compared to BFM II, with a marked difference observed (24/31, 0.774 vs. 9/21, 0.429, *p* = 0.011 < 0.05), as shown in Figure 3ii.

In our research endeavors, we sought to identify factors associated with BFM. Significant disparities were noted in the prevalence of prior cerebral hemorrhage, as illustrated in Table 3, where the incidence within BFM II was observed to exceed that of BFM I. Under the hypothesis that BFM II was in a relatively less ischemic state compared to BFM I, we speculated that hemispheres which have experienced cerebral hemorrhage may be in a relatively non-ischemic state compared to other MMD patients. But further investigation was warranted to substantiate this hypothesis. When comparing the postoperative mRS scores between BFM I and BFM II, no significant differences were observed, indicating no distinct difference in the ability to improve patients’ symptoms. Out of the 118 hemispheres with s-s bypass, only 40 underwent repeat DSA after operation, representing a relatively small sample size. Despite the potential for error due to the limited number of cases, no obvious differences were noted in Matsushima grade between the two groups, indirectly suggesting no difference in revascularization ability in terms of long-term prognosis, as illustrated in Figure 5i.

Long-standing ischemia can impair the self-regulation of blood vessels in the affected area, potentially leading to a loss of the ability of intracranial vessels to reflexively constrict when faced with blood flow from extracranial sources. During cerebral ischemia and reperfusion, the release of nitric oxide, oxygen free radicals, various inflammatory factors, and chemokines may further impact the self-regulation of cerebral blood vessels, potentially damaging the blood-brain barrier and leading to the occurrence of CHS (4, 5, 17–19). This may be the reason why CHS still occurred despite the less blood flowing into the intracranial in BFM II.

Our prior research indicated that s-s anastomosis was significantly more effective in reducing cerebral hyperperfusion (CHP) compared to e-s anastomosis (20). However, in the current study, no difference was observed in the incidence of CHS between the two methods. This discrepancy may stem from the difference between CHS and CHP and the varying proportions of blood flow models in the patient population. In our analysis, BFM I was prevalent, representing a substantial portion of the cases (65/118, 55.1%). This prevalence could be a factor in the lack of difference between s-s and e-s bypass observed in this study. A closer examination of the data suggested that the incidence of CHS in BFM I was marginally higher than in e-s bypass (0.244 vs. 0.229), although no significant difference was identified. Additionally, the symptoms in BFM I appeared to be more severe, potentially due to BFM I’s propensity to draw a greater volume of blood into the intracranial space than e-s bypass. Further data was required to substantiate these observations.

The analysis between the e-s bypass and BFM II in s-s bypass demonstrated that BFM II had a lower incidence of CHS compared to the traditional e-s bypass. Notably, in our study, CHS was observed in only one patient with BFM II out of 53 cases (1.9%). This lower incidence of CHS in BFM II may be attributed to the less severe cerebral ischemia, where the self-regulation and reactivity of cerebral blood vessels remained relatively intact, thus offering significant adaptability in response to extracranial blood flow. Additionally, the presence of a shunt through the distal STA in BFM II allowed for a reduced volume of blood entering the intracranial space, which in turn contributes to the decreased incidence of CHS. This suggested that the preservation of the distal STA in s-s bypass might play a crucial role in modulating blood flow and preventing excessive perfusion, thereby potentially lowering the risk of CHS.

In clinical practice, for patients who have undergone s-s bypass and are diagnosed with BFM I, there should be heightened vigilance regarding blood pressure management and the volume of intravenous fluid supplementation to prevent the occurrence of CHS. Moreover, discovering strategies to convert all postoperative blood flow models into BFM II, or to reduce the prevalence of BFM I, could significantly lower the incidence of CHS following s-s anastomosis in patients with MMD.

### Limitations

A notable limitation of this study was its nature as a single-center, retrospective analysis involving a modest number of patients. We utilized a basic qualitative approach to define CHS and the degree of ischemia, which might have overlooked numerous quantitative data points, potentially leading to inaccuracies in our findings. The retrospective design of the study resulted in small subgroup sizes, which could have affected the outcomes. In terms of BFM formation, we only speculated on its correlation with the severity of ischemia, while the intraoperative factors, such as the diameters of the donor and recipient blood vessels, the dimensions of the anastomotic stoma, and blood flow velocities, were not examined due to the retrospective methodology of the study. Due to the limitations of our study’s single-center nature and the fact that the bypass surgery was performed by only one surgeon, further research with larger cohorts and prospective data collection was needed to validate and expand upon these findings.

## Conclusion

The incidence of postoperative CHS in BFM II during s-s bypass was found to be lower than that in e-s bypass and BFM I within the s-s bypass. The BFM could potentially emerge as a new predictive indicator for the occurrence of CHS after s-s bypass surgery.

### Prospect

In the context of s-s bypass surgery for MMD, we believe that the next pivotal research endeavor should be focused on identifying methods to consistently direct all blood flow models towards the more favorable BFM II or to reduce the incidence of BFM I. Achieving this could significantly lower the occurrence of CHS following s-s bypass in MMD patients.

## Data Availability

The raw data supporting the conclusions of this article will be made available by the authors, without undue reservation.

## Supplementary material

SUPPLEMENTARY VIDEO S1 Demonstration of BFM I in ICG-VA. The video illustrates the characteristic blood flow fluctuations observed around the anastomosis site and the distal STA, where a significant delay in blood flow is evident, indicative of BFM I.

SUPPLEMENTARY VIDEO S2 Demonstration of BFM II in ICG-VA. This video captures the blood flow from the proximal STA moving unimpeded into the distal STA, showcasing the unobstructed flow pattern that defines BFM II.

## References

[1] Zhang X Xiao W Zhang Q Xia D Gao P Su J. Progression in moyamoya disease: clinical features, neuroimaging evaluation, and treatment. Curr Neuropharmacol. (2022) 20:292–308. doi: 10.2174/1570159x19666210716114016, PMID: 34279201 PMC9413783

[2] Ihara M Yamamoto Y Hattori Y Liu W Kobayashi H Ishiyama H. Moyamoya disease: diagnosis and interventions. Lancet Neurol. (2022) 21:747–58. doi: 10.1016/s1474-4422(22)00165-x 35605621

[3] Donaghy RM Yasargil G. Microangeional surgery and its techniques. Prog Brain Res. (1968) 30:263–7. doi: 10.1016/s0079-6123(08)61469-7 5735457

[4] Fujimura M Kaneta T Mugikura S Shimizu H Tominaga T. Temporary neurologic deterioration due to cerebral hyperperfusion after superficial temporal artery-middle cerebral artery anastomosis in patients with adult-onset moyamoya disease. Surg Neurol. (2007) 67:273–82. doi: 10.1016/j.surneu.2006.07.017, PMID: 17320638

[5] Lin YH Liu HM. Update on cerebral hyperperfusion syndrome. J Neurointerv Surg. (2020) 12:788–93. doi: 10.1136/neurintsurg-2019-015621, PMID: 32414892 PMC7402457

[6] Zhang X Ni W Feng R Li Y Lei Y Xia D. Evaluation of hemodynamic change by indocyanine green-FLOW 800 Videoangiography mapping: prediction of hyperperfusion syndrome in patients with moyamoya disease. Oxid Med Cell Longev. (2020) 2020:8561609. doi: 10.1155/2020/8561609, PMID: 32850003 PMC7441439

[7] Singh R McLelland MD De La Peña NM Pollock JR Catapano JS Srinivasan VM. Research advances in the diagnosis and treatment of moyamoya disease: a bibliometric analysis. Neurosurg Rev. (2022) 45:1977–85. doi: 10.1007/s10143-022-01748-w, PMID: 35150354

[8] Shi Z Wu L Wang Y Li W Wang J Yang Y. Intraoperative hemodynamics of parasylvian cortical arteries for predicting postoperative symptomatic cerebral hyperperfusion after direct revascularization in patients with moyamoya disease: a preliminary study. J Clin Med. (2023) 12:–3855. doi: 10.3390/jcm12113855 PMC10253873 37298050

[9] Ogasawara K Konno H Yukawa H Endo H Inoue T Ogawa A. Transcranial regional cerebral oxygen saturation monitoring during carotid endarterectomy as a predictor of postoperative hyperperfusion. Neurosurgery. (2003) 53:309–15. doi: 10.1227/01.neu.0000073547.86747.f3 12925245

[10] Lu L Huang Y Han Y Li Y Wan X Chen J. Clinical effect of a modified superficial temporal artery-middle cerebral artery bypass surgery in Moyamoya disease treatment. Front Neurol. (2023) 14:1273822. doi: 10.3389/fneur.2023.1273822, PMID: 37941571 PMC10628485

[11] Khan NR Lu VM Elarjani T Silva MA Jamshidi AM Cajigas I. One-donor, two-recipient extracranial-intracranial bypass series for moyamoya and cerebral occlusive disease: rationale, clinical and angiographic outcomes, and intraoperative blood flow analysis. J Neurosurg. (2022) 136:627–36. doi: 10.3171/2021.2.Jns204333, PMID: 34416732

[12] Cikrit DF Burt RW Dalsing MC Lalka SG Sawchuk AP Waymire B. Acetazolamide enhanced single photon emission computed tomography (SPECT) evaluation of cerebral perfusion before and after carotid endarterectomy. J Vasc Surg. (1992) 15:747–54. doi: 10.1016/0741-5214(92)90708-G 1578529

[13] Zhang J Yu J Xin C Fujimura M Lau TY Hu M. A flow self-regulating superficial temporal artery-middle cerebral artery bypass based on side-to-side anastomosis for adult patients with moyamoya disease. J Neurosurg. (2023) 138:1347–56. doi: 10.3171/2022.8.Jns221379, PMID: 36461841

[14] Lang MJ Kan P Baranoski JF Lawton MT. Side-to-Side Superficial Temporal Artery to Middle Cerebral Artery Bypass Technique: Application of Fourth Generation Bypass in a Case of Adult Moyamoya Disease. Oper Neurosurg (Hagerstown). (2020) 18:480–486. doi: 10.1093/ons/opz268, PMID: 31768535

[15] Research Committee on the Pathology and Treatment of Spontaneous Occlusion of the Circle of Willis, Health Labour Sciences Research Grant for Research on Measures for Intractable Diseases. Guidelines for diagnosis and treatment of moyamoya disease (spontaneous occlusion of the circle of Willis). Neurol Med Chir. (2012) 52:245–66. doi: 10.2176/nmc.52.245, PMID: 22870528

[16] Uda K Araki Y Muraoka S Ota S Wada K Yokoyama K. Intraoperative evaluation of local cerebral hemodynamic change by indocyanine green videoangiography: prediction of incidence and duration of postoperative transient neurological events in patients with moyamoya disease. J Neurosurg. (2018) 130:1367–75. doi: 10.3171/2017.10.Jns171523, PMID: 29676693

[17] Yoon HK Oh H Lee HC Cho WS Kim JE Park JW. Effect of sevoflurane postconditioning on the incidence of symptomatic cerebral hyperperfusion after revascularization surgery in adult patients with moyamoya disease. World Neurosurg. (2020) 134:e991–e1000. doi: 10.1016/j.wneu.2019.11.055, PMID: 31734419

[18] Narducci A Yasuyuki K Onken J Blecharz K Vajkoczy P. *In vivo* demonstration of blood-brain barrier impairment in moyamoya disease. Acta Neurochir. (2019) 161:371–8. doi: 10.1007/s00701-019-03811-w, PMID: 30675657

[19] Fujimura M Mugikura S Kaneta T Shimizu H Tominaga T. Incidence and risk factors for symptomatic cerebral hyperperfusion after superficial temporal artery-middle cerebral artery anastomosis in patients with moyamoya disease. Surg Neurol. (2009) 71:442–7. doi: 10.1016/j.surneu.2008.02.031, PMID: 18514264

[20] Tao T Zhu W Yu J Li X Wei W Hu M. Intraoperative evaluation of local cerebral hemodynamic change by laser speckle contrast imaging for predicting postoperative cerebral hyperperfusion during STA-MCA bypass in adult patients with moyamoya disease. J Cereb Blood Flow Metab. (2024) 44:1163–73. doi: 10.1177/0271678x241226483 38233750 PMC11179619

